# Editorial: Interactions of Pentraxins and Complement in Infection, Inflammation, and Cancer

**DOI:** 10.3389/fimmu.2022.861359

**Published:** 2022-02-17

**Authors:** Ying Jie Ma, Andrea Doni, Cecilia Garlanda

**Affiliations:** ^1^ Department of Clinical Immunology, Rigshospitalet, Faculty of Health and Medical Sciences, University of Copenhagen, Copenhagen, Denmark; ^2^ Department of Inflammation and Immunology, Humanitas Clinical and Research Center IRCCS, Milan, Italy; ^3^ Department of Biomedical Sciences, Humanitas University, Pieve Emanuele, Italy

**Keywords:** pentraxins, complement, infection, inflammation, cancer, PTX3, CRP, SAP

Complement is an important innate immune defense system that deploys efficient immuno-surveillance and protection against pathogens and damaged host cells, and plays crucial roles in inflammation (1). The complement comprises over 50 effectors and receptors, which precisely balance activation, amplification and regulation of the complement proteolytic cascade organized by three canonical pathways (2). However, the complement often becomes host-offensive and leads to pathophysiological situations once disordered (3).

Pentraxins are a superfamily of conserved multi-functional pattern recognition molecules (PRMs) with features of a cyclic multimeric structure and a conserved C-terminal pentraxin domain. As major members of the pentraxin family, C-reactive protein (CRP), serum-amyloid P component (SAP) and pentraxin 3 (PTX3) are synthesized mainly in response to inflammatory mediators and tissue injury, and function as essential constituents in innate immune defense against certain pathogenic intruders (4). Featuring antibody-like common properties, CRP, SAP and PTX3 often induce rapid and efficient immune effector mechanisms upon selective recognition of microbial moieties (5). Interestingly, recent data have shown that interaction between pentraxins and complement could build up comprehensive crosstalk with the three pathways of complement cascade activation (6).

This Research Topic aims at collecting the latest findings to gain deeper insight into the functional roles of pentraxins in infection and regulation of inflammatory responses with a focus on the interaction with the complement system, thus directing potential therapeutic applications.

Both pentraxins and complement play a non-redundant role in resistance to invasive pulmonary aspergillosis caused by *Aspergillus fumigatus* (*A. fumigatus*), but their crosstalk also has a synergic effect on their immune effector functions. Dellière et al. dissect the regulation of alveolar humoral immune components during *A. fumigatus* infection (7). Using comparative proteomic analysis of the bronchoalveolar lavage (BAL) fluid collected from individuals infected or colonized with *A. fumigatus*, they identified several humoral immune components affected by *A. fumigatus* infection and colonization. In agreement with previous findings, both complement factors and pentraxins were found as the major humoral immune components, for instance C1q, ficolin-2, MASP-2 and PTX3. These findings further substantiate an essential contribution of these PRMs and their synergistic effects *via* crosstalk during *A. fumigatus-*caused infection and inflammation. Further, the review article by Parente et al. highlights the roles of pentraxins in the complement-mediated immune response to *A. fumigatus* infection (8). In particular, in light of an emerging antifungal role of SAP (9), the authors discussed how the complement forms an integrated system by means of crosstalk, synergism and regulation with the pentraxins, thus reinforcing efficacy of fungal pathogen recognition and clearance.

CRP is an acute phase reactant produced in response to inflammatory mediators associated with damage and inflammatory conditions in human. An emerging characteristic of CRP is the modulation of its functional activities through conformational changes under pathophysiological situations. Noone et al. report their latest findings using high-resolution cryo-electron microscopy and ELISA showing how pH and ligand binding affect the structure and biochemical properties of CRP (10). Compared with previous CRP structure analysis studied under physiological pH by crystallography, this study applied single-particle analysis cryoEM to solve the first solution-phase structures of CRP at acidic pH and in the presence of ligands. The study further substantiates that CRP increases its capacity of association specifically with complement initiating factors under acidic pH, which typically prevails at local site of acidosis, demonstrating that this occurs through a conformational switch. A pH-dependent switch-on effect has also been described for PTX3 in tissue repair (11). These studies demonstrate the relevance of specific tissue microenvironment conditions in pentraxin biologic activity.

Pentraxins selectively recognize pathogens including bacteria, fungi and virus, and efficiently remove the opsonized pathogens through phagocytosis. This can be achieved in a direct fashion or in an indirect fashion *via* heterocomplex formation with complement-related PRMs against a wide spectrum of pathogen-associated molecular patterns (PAMPs). The research by Asgari et al. describes the contribution of PTX3 in resistance against *Klebsiella pneumoniae* (*K. pneumoniae*) infection (12). Using mouse models of severe *K. pneumoniae* pulmonary infection, Asgari et al. found that PTX3-deficiency was associated with increased susceptibility to *K. pneumoniae* infection, higher bacterial load, mortality and inflammation, and demonstrate that locally and systemically upregulated PTX3 restricts the infection through modulation of inflammatory responses and tissue damage. However, the protective roles of PTX3 was independent of opsonophagocytosis or complement activation and regulation, suggesting the relevance of PTX3-dependent regulation of inflammation and tissue damage in innate responses to certain infections.

Using two pathogenic *Sporothrix* species, *Sporothrix schenckii* (*S. schenckii*) and *Sporothrix brasiliensis* (*S. brasiliensis*), as model of infections, Neves et al. identified a unique PAMP on the cell wall of *Sporothrix* species involved in complement-dependent immune responses (13). They found that cell wall peptidorhamnomannan is responsible for mediating phagocytosis and inflammatory cytokine expression against challenge of *Sporothrix* species through opsonization and activation of complement receptor 3 (CR3) in a complement-dependent fashion. NMR analysis revealed differences of peptidorhamnomannan in the cell wall structure and content of rhamnose in the two *Sporothrix* species, resulting in differential pathways of complement activation. Interestingly, the cell wall peptidorhamnomannan was also found to stimulate PTX3 secretion by monocyte-derived macrophage challenged with *Sporothrix* in the presence of complement factors, suggesting the relevance of the complement system in phagocyte inflammatory responses to the causative agents of human and animal sporotrichosis.

In summary, this Research Topic has brought to light emerging new findings about the interaction between pentraxins and complement and its pivotal roles in infection and inflammation. The pivotal link between pentraxins and complement has been increasingly highlighted for their roles in immunosurveillance in cancer, innate immune resistance against infection and regulation of inflammation. However, whether this crosstalk is associated with immunopathology during infection and inflammation and how it exacerbates disease severity are also intriguing questions to be answered. Therefore, we anticipate that sustainable research will make this field flourishing over the upcoming years for novel immunotherapeutic strategies.

## Author Contributions

All authors have made a substantial, direct and intellectual contribution to the work. YM drafted the manuscript. The other editors AD and CG provided critical revisions and approved the submitted manuscript.

## Funding

Associazione Italiana Ricerca sul Cancro (AIRC IG-21714 to CG); Ministero della Salute (RF-2013-02355470 to CG); Ministero dell’Istruzione, dell’Università e della Ricerca (MIUR) (PRIN 20174T7NXL to CG).

## Conflict of Interest

The authors declare that the research was conducted in the absence of any commercial or financial relationships that could be construed as a potential conflict of interest.

## Publisher’s Note

All claims expressed in this article are solely those of the authors and do not necessarily represent those of their affiliated organizations, or those of the publisher, the editors and the reviewers. Any product that may be evaluated in this article, or claim that may be made by its manufacturer, is not guaranteed or endorsed by the publisher.
